# Conservative Management of Subluxation Injuries in Young Permanent Teeth Using Fiber-Reinforced Composite Splints: Review and Case Reports

**DOI:** 10.7759/cureus.105025

**Published:** 2026-03-11

**Authors:** Mridula Goswami, Vashi Narula, Vishal Vishal

**Affiliations:** 1 Department of Pediatric and Preventive Dentistry, Maulana Azad Institute of Dental Sciences, New Delhi, IND

**Keywords:** fiber reinforced composite splint, flexible splinting, permanent teeth, tooth subluxation, traumatic dental injuries

## Abstract

Subluxation of young permanent teeth poses a significant clinical challenge due to its potential impact on pulp vitality, periodontal healing, and esthetics. Luxation injuries in anterior teeth are more common in children and adolescents, often resulting from sports injuries, falls, or accidents. Management of these injuries requires accurate diagnosis, prompt repositioning, and effective stabilization. Fiber-reinforced composite (FRC) splints, being flexible and esthetic, have emerged as a preferred option in anterior teeth stabilization in pediatric and adolescent patients.

This case series highlights the diagnosis and management of subluxation injury in three pediatric patients aged 6-12 years, each presenting with varying forms of subluxation injury of young permanent incisors. Teeth were repositioned and stabilized with FRC splints following the International Association of Dental Traumatology (IADT) 2020 guidelines. Clinical and radiographic follow-ups demonstrated favourable healing outcomes with no evidence of adverse sequelae.

Fiber-reinforced composite splints represent a clinically predictable, minimally invasive, and esthetic alternative to wire splints in the management of subluxation injuries of anterior teeth in adolescents. Their flexibility and stabilization efficacy align with IADT recommendations, promoting periodontal healing and patient satisfaction.

Fiber-reinforced composite splints can be considered the splint of choice for managing luxation injuries in young permanent teeth, particularly in esthetically sensitive zones of adolescents, where functional and psychosocial outcomes are equally important.

## Introduction

Traumatic dental injuries (TDIs) represent a significant global public health concern in children. Often these result from falls, sports activities, and accidental impacts. The global prevalence of TDIs in permanent dentition is reported to be 19.5%, whereas in India, the prevalence is 12% among those older than 6 years [1,2]. Luxation injuries constitute one of the most frequently encountered categories of TDIs in children and adolescents, reflecting the high susceptibility of the periodontal ligament to impact forces [3]. Subluxation, defined as an injury to the periodontal tissues resulting in increased tooth mobility without displacement, is among the most common luxation injuries, accounting for 15-61% of TDIs in permanent teeth [4]. Such injuries result from disruption of the periodontal ligament fibres and the neurovascular supply to the pulp, leading to inflammatory responses within the periodontal ligament and pulpal tissues. Damage to these supporting structures may compromise pulpal blood flow and cellular viability, potentially resulting in pulpal necrosis, inflammatory root resorption, or impaired periodontal healing if not appropriately managed.

Recognizing the importance of standardized protocols, the International Association of Dental Traumatology (IADT) has revised evidence-based guidelines for the management of subluxation in young permanent teeth. Management of subluxation primarily focuses on relieving symptoms, monitoring pulpal status, and stabilizing the traumatized tooth when mobility or patient discomfort is significant. According to IADT 2020 guidelines, subluxated teeth may be managed either without splinting or with flexible splinting for up to 2 weeks when mobility is pronounced or painful [3]. Various splints satisfying these parameters include acid-etch composite with stainless-steel wire (≤0.4 mm), titanium trauma splints (TTS), nylon fishing line splints, orthodontic elastics, and resin-based flexible splints [5]. All these are designed to maintain tooth position while allowing controlled physiological mobility [6].

Flexible splints permit limited physiological tooth movement, which helps maintain functional stimulation of the periodontal ligament and promotes organized fibre healing while reducing the risk of complications such as ankylosis or replacement resorption that may occur with rigid immobilization. Among these options, fiber-reinforced composite (FRC) splints have emerged as one of several flexible splinting approaches because of their favorable esthetics, adaptability, and adequate flexural properties. FRC splints consist of polyethylene or glass fibers embedded in a resin matrix, offering strength, translucency, and ease of application, but they require adequate moisture control and technique sensitivity during bonding and may exhibit plaque accumulation if not contoured properly [7]. Although IADT does not mandate a specific splint type, FRC splints fall within the recommended category of flexible, passive, short-term splints, making them a clinically acceptable alternative to wire-composite and titanium splints, which have long been used for managing subluxation injuries.

This case series aims to describe the clinical presentation, management, and short-term outcomes of subluxation injuries in young permanent anterior teeth stabilized using fiber-reinforced composite splints in accordance with IADT 2020 guidelines.

## Case presentation

The present case series describes three patients, aged between 6 and 12 years, who reported to the emergency department of Pediatric and Preventive Dentistry with subluxation injury to young permanent anterior teeth. For each case, a comprehensive history was recorded, including demographic details as well as past medical and dental history. The medical history of all patients was insignificant. Patients had suffered from trauma with no reported history of loss of consciousness, vomiting, seizures, or ear, nose, or throat bleeding; however, a history of oral bleeding was present.

Patients aged 6-12 years who reported to the emergency department with TDIs with subluxation of immature permanent incisors with Miller’s Grade I or Grade II mobility, intact crown structure suitable for bonding, and the ability to comply with scheduled follow-up visits were included in this case series. Whereas patients with extensive crown loss preventing splint placement, associated alveolar fractures requiring rigid immobilization, systemic medical conditions affecting healing, or inability to maintain follow-up were excluded.

The present case series was conducted in accordance with the ethical principles outlined in the Declaration of Helsinki (World Medical Association, 2013 revision) [8]. Written informed consent was obtained from the parents or legal guardians before treatment and documentation of the cases.

Tooth numbering in this report follows the Fédération Dentaire Internationale (FDI) two-digit system for identification of individual teeth [9]. In all cases, comprehensive dental trauma management was carried out, including stabilization of teeth using fiber-reinforced composite splinting in accordance with the IADT guidelines 2020. All patients were scheduled for regular clinical and radiographic follow-up to monitor healing and treatment outcomes at 1 week, 2 weeks, 4 weeks, 3 months, and 6 months, during which additional preventive and restorative procedures such as composite restorations and pit and fissure sealant applications were also performed as part of comprehensive pediatric dental care. Accordingly, the management of subluxation injuries must follow the IADT 2020 guidelines, which are summarized in Table 1 for clarity and uniform clinical application [3].

**Table 1:** IADT 2020 guidelines for subluxation This is an original compilation by the authors based on Bourguignon et al. [3]. IADT: International Association of Dental Traumatology

Parameter	IADT 2020 Recommendation
Clinical Findings	Increased mobility of the tooth with or without displacement, tender to biting/percussion, gingival sulcus bleeding, and possible transient loss of sensibility response.
Radiographic Findings	No evident findings; recommended radiographs: one periapical view, two additional angled views, occlusal radiograph.
Treatment	No active treatment in grade I mobility; a passive, flexible splint may be placed for up to 2 weeks only if mobility is excessive or painful.
Follow-up Schedule	2 weeks (splint removal, if placed), 12 weeks, 6 months, 1 year, then annually for at least 5 years.
Favorable Outcomes	Asymptomatic, positive sensibility response (may be delayed), intact lamina dura, continued root development in immature teeth.
Unfavorable Outcomes	Pulp necrosis, apical periodontitis, lack of root development (in immature teeth), or development of external inflammatory resorption requiring immediate endodontic intervention.

Case 1

An 8-year-old male patient reported a history of trauma to the maxillary anterior teeth due to a road traffic accident 10 days before presentation. Primary treatment had been provided at the reporting hospital for pain management.

**Figure 1:**
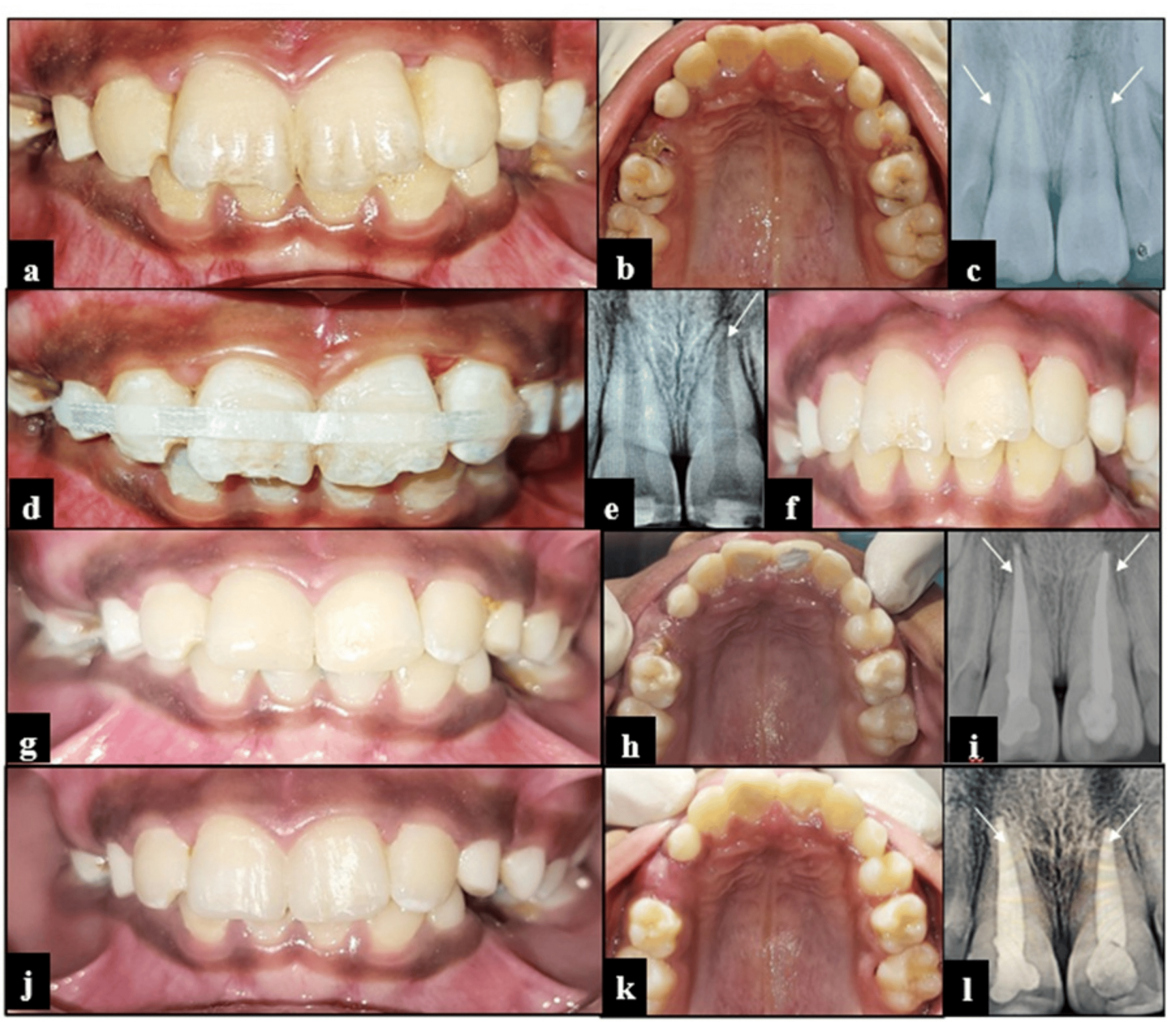
Management of subluxated maxillary incisors using fiber-reinforced composite splinting in Case 1 (a,b) Pre-operative frontal and maxillary occlusal views showing enamel fracture with respect to 11 and subluxation with respect to 11 and 21; (c) Pre-operative RVG, wherein 11 and 21 show periodontal ligament space widening; (d) Post-operative frontal view with FRC splinting; (e) Post-operative RVG with respect to 11 and 21 of  FRC splinting; (f) Frontal view after splint removal at the two-week follow-up; (g-i) Root canal treatment, followed by composite restoration with respect to 11 and 21 at the two-week follow-up; (j-l) Frontal view, maxillary occlusal view and RVG of 11 and 21 at the six-month follow-up. RVG: radiovisiography; FRC: fiber-reinforced composite

Intraoral examination revealed Ellis Class I fracture and subluxation of teeth 11 and 21 (Fig. 1a, 1b). Radiovisiography (RVG) of 11 and 21 demonstrated periodontal ligament (PDL) space widening without periapical pathology (Fig. 1c). On clinical reassessment, Miller’s grade II mobility was observed in both teeth 11 and 21. Due to aesthetic concerns, a fiber-reinforced composite splint was planned (Kids-e-Dental E-Splint, Mumbai, India) involving teeth 53, 12, 11, 21, 22, and 63 (Fig. 1d, 1e). At the one-week follow-up, the patient reported pain in relation to tooth 11. Pulp vitality testing using cold test (Endo-Ice, Coltene, Altstätten, Switzerland) was non-responsive, and the tooth was tender to percussion. RVG of tooth 11 showed a closed apex with a periapical radiolucency having a Periapical Index (PAI) score of 3, along with evidence of external root resorption in tooth 21. Consequently, root canal treatment was done on 11 and 21, followed by composite restoration. At the two-week follow-up, the splint was removed (Fig. 1f). Both teeth demonstrated satisfactory clinical and radiographic healing with no abnormal mobility (Fig. 1g-1i). Clinical and radiographic assessments conducted during the three-month and six-month follow-ups demonstrated satisfactory healing outcomes, with no evidence of adverse sequelae such as periapical pathology or abnormal mobility (Figure 1j-1l).

Case 2

A 6-year-old male patient presented with a history of trauma to maxillary and mandibular anterior teeth sustained during play one day before presentation.

**Figure 2:**
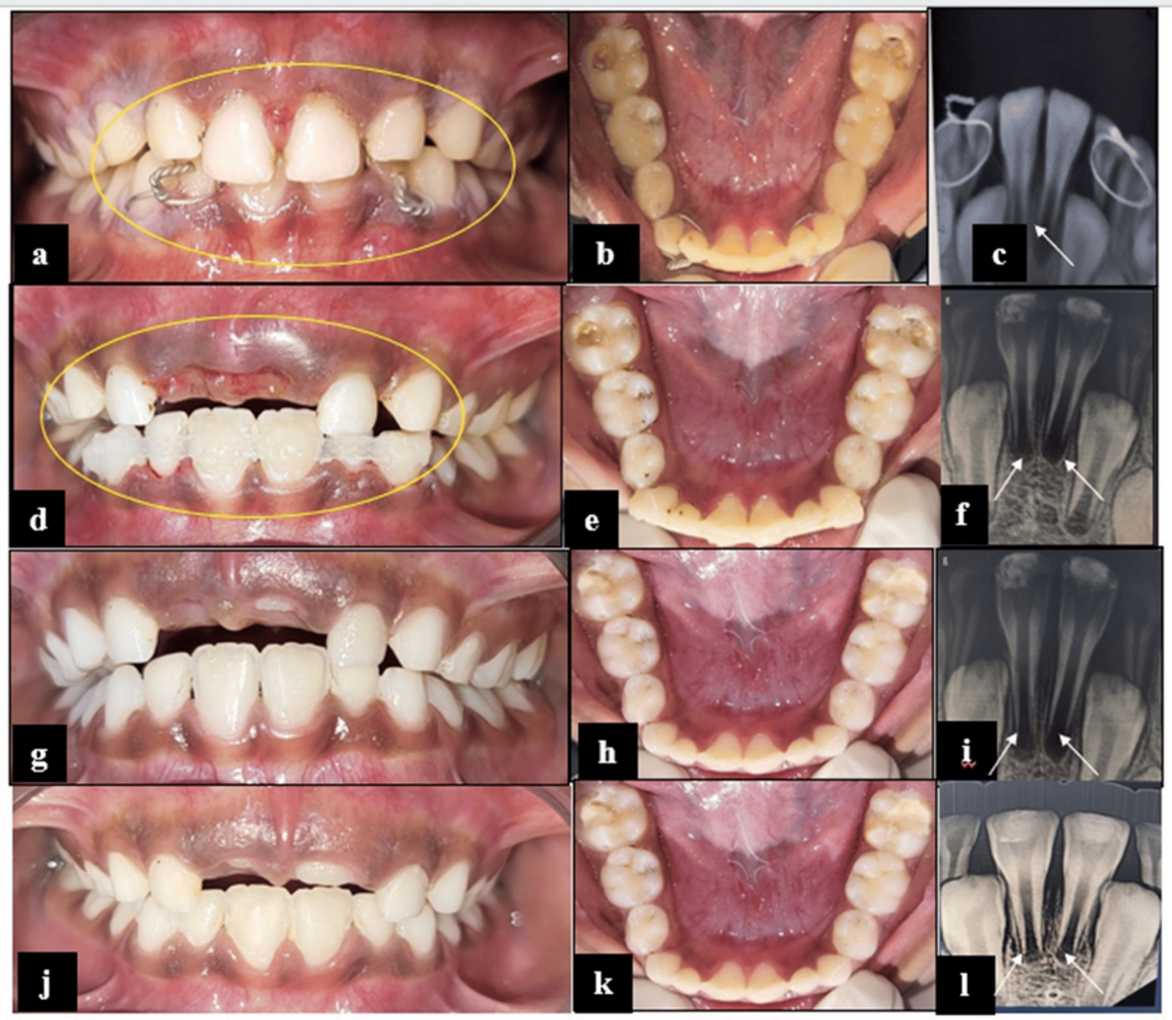
Management of subluxated mandibular incisors using fiber-reinforced composite splinting in Case 2 (a,b) Pre-operative frontal and mandibular occlusal views showing extrusive luxation of 51 and 61, and bridle wiring of 82, 41, 31, and 72; (c) Pre-operative RVG of 82, 41, 31, and 72; (d) Post-operative frontal view after extraction of 51 and 61; (e) Post-operative mandibular occlusal view with FRC splinting; (f) Post-operative RVG of FRC splinting; (g-i) Frontal, mandibular occlusal view and RVG of 82, 41, 31, and 72 after splint removal at the two-week follow-up; (j-l) Frontal view, mandibular occlusal view, and RVG of 82, 41, 31, and 72 at the six-month follow-up. RVG: radiovisiography; FRC: fiber-reinforced composite

Intraoral examination revealed extrusive luxation of teeth 51 and 61, and subluxation of teeth 82, 41, 31, and 72 (Fig. 2a). Initial emergency management of the trauma had already been performed, wherein bridle wiring was placed for stabilization of the subluxated teeth (Fig. 2b). RVG of 51 and 61 demonstrated physiologic root resorption, while RVGs of the 83, 82, 41, 31, 72, and 73 regions confirmed the presence of bridle wiring in situ extending from 83 to 82 and from 72 to 73 (Fig. 2c). On clinical reassessment, Miller’s grade II mobility was observed with respect to teeth 72 31, 41, and 82. A definitive treatment plan was formulated, which included the extraction of mobile primary teeth 51 and 61 under local anesthesia, followed by the removal of bridle wiring in lower teeth. Fiber-reinforced composite splinting (Kids-e-Dental E-Splint, Mumbai, India) was performed involving teeth 83, 82, 41, 31, 72, and 73 for stabilization of subluxated teeth (Fig. 2d-2f). At the two-week follow-up, no mobility was observed in the splinted teeth. Accordingly, the splint was removed, and satisfactory healing was noted (Fig. 2g-2i). Clinical and radiographic assessments conducted during the three-month and six-month follow-ups demonstrated satisfactory healing outcomes, with no evidence of adverse sequelae (Fig. 2j-2l).

Case 3

An 11-year-old male patient presented with a history of trauma to the maxillary anterior teeth due to a fall while playing, 10 days before presentation. Primary treatment had been provided at the reporting hospital for pain management.

**Figure 3:**
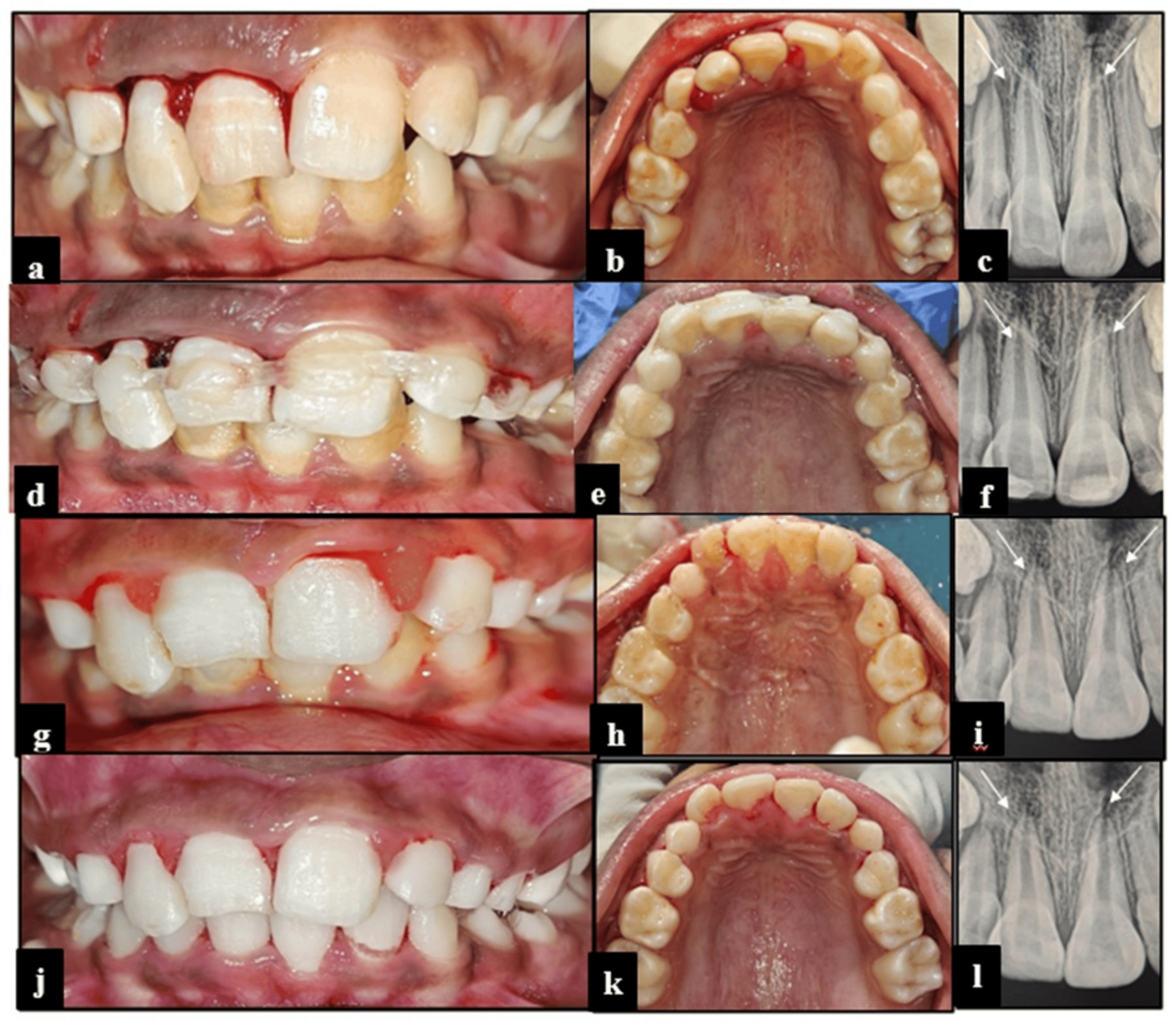
Management of subluxated and extruded maxillary incisors using fiber-reinforced composite splinting in Case 3 (a,b) Pre-operative frontal and maxillary occlusal views showing subluxation of 11 and 21 and extrusion of 12; (c) Pre-operative RVG, wherein 11, 12, and 21 show periodontal ligament space widening; (d,e) Post-operative frontal and maxillary occlusal view with FRC splinting; (f) Post-operative RVG of 11, 12, and 21 of FRC splinting; (g-i) Frontal view, maxillary occlusal view, and RVG of 11, 12, and 21 after splint removal at the two-week follow up; (j-l) Frontal, maxillary and occlusal views and RVG of 11, 12, and 21 at the six-month follow-up. RVG: radiovisiography; FRC: fiber-reinforced composite

Intraoral examination revealed subluxation of teeth 11 and 21, and extrusion of tooth 12 (Fig. 3a, 3b). RVG of 12, 11, and 21 demonstrated PDL space widening without periapical pathology (Fig. 3c). On clinical reassessment, Miller’s grade II mobility was observed with respect to teeth 12, 11, and 21. The definitive treatment plan included repositioning of tooth 12, followed by fiber-reinforced composite splinting (Kids-e-Dental E-Splint, Mumbai, India) involving teeth 53, 12, 11, 21, 22, and 63 for the stabilization of the subluxated and repositioned teeth (Fig. 3d-3f). At the two-week follow-up, no abnormal mobility was observed in the splinted teeth. Accordingly, the splint was removed, and satisfactory healing was noted (Fig. 3g-3i). Clinical and radiographic assessments conducted during the three-month and six-month follow-ups demonstrated satisfactory healing outcomes, with no evidence of adverse sequelae (Fig. 3j-3l).

Steps of fiber-reinforced composite splinting

The clinical procedure for fiber-reinforced composite (FRC) splinting was performed according to the steps in Table 2 [7].

**Table 2:** Steps of fiber-reinforced composite splinting This is an author-compiled summary based on Strassler and Serio [7].

Steps	Procedure
Initial Preparation	A thorough assessment was done, followed by debridement (if required) and repositioning of any displaced teeth, while achieving hemostasis of the area using cotton rolls or gauze.
Surface Treatment	The labial surfaces of teeth to be splinted were cleaned and etched using 35–37% phosphoric acid for 15–20 seconds.
Rinsing and Drying	Rinsing of the etched surfaces was performed for 10 seconds, followed by gentle air-drying for 5 seconds.
Bonding	Application of a bonding agent was done, followed by light-curing for 20 seconds.
Fiber Adaptation	The fiber ribbon (Kids-e-Dental E-Splint, Mumbai, India) was measured, cut to the required length, and adapted along the middle third of the labial surfaces of teeth to be splinted.
Fixation	Flowable composite resin was applied, and the fiber ribbon was positioned and light-cured at each tooth, followed by coverage of the assembly with a thin layer of composite resin.
Finishing	Occlusion was checked to eliminate any interferences, and the margins were finished to enhance patient comfort.
Post-operative Care	Post-operative instructions were given to the patient, including maintenance of a soft diet, reinforcement of oral hygiene practices, and compliance with scheduled follow-up visits.
Removal and Reassessment	The splint was removed after a period of two weeks, after which tooth mobility and pulpal status were carefully reassessed clinically and radiographically at subsequent follow-up visits.

## Discussion

Traumatic dental injuries in young permanent teeth require careful clinical judgment, as their prognosis depends largely on prompt diagnosis, appropriate repositioning, and adequate stabilization. In the present case series, three young male patients with varying degrees of subluxation injuries were managed using fiber-reinforced composite (FRC) splints, following IADT 2020 guidelines. The consistent clinical and radiographic outcomes observed across all cases underline the effectiveness of flexible splinting in achieving periodontal and pulpal healing in growing adolescents. To facilitate comparison of the diagnostic characteristics and management of the presented cases, a standardized summary based on IADT 2020 criteria is provided in Table 3.

**Table 3:** Standardized diagnostic and clinical characteristics of the presented cases based on IADT 2020 criteria IADT:  International Association of Dental Traumatology; FRC: fiber-reinforced composite; PDL: periodontal ligament

Parameter	Case 1	Case 2	Case 3
Patient Age	8 years	6 years	11 years
Time Since Trauma	10 days	1 day	10 days
Teeth Involved	11, 21	51, 61 (extrusion); 82, 41, 31, 72 (subluxation)	12 (extrusion); 11, 21 (subluxation)
Mobility Grade (Miller’s Grading)	Grade II (11, 21)	Grade II (82, 41, 31, 72)	Grade II (12, 11, 21)
Displacement	None (subluxation)	Extrusion of primary maxillary central incisors; no displacement in permanent mandibular central incisors	Extrusion of 12; no displacement of 11 and 21
Sensibility Testing (Baseline)	No response in 11 and 21	Normal response in 41 and 31	Normal response in 11 and 21, delayed response in 12
Percussion Sound	Normal	Normal	Normal
Radiographic Findings	Widened PDL space; no initial periapical pathology	Physiologic root resorption (51 and 61); no pathology in permanent teeth	PDL widening; no periapical pathology
Root Development	Complete (closed apices) in 11 and 21	Open apices in 31 and 41	Complete (closed apices) in 11 and 21
Final Diagnosis (IADT 2020)	Subluxation of 11 and 21	Subluxation of 82, 41, 31, and 72; extrusive luxation of 51 and 61	Extrusive luxation of 12; subluxation of 11 and 21
Treatment Provided	FRC splinting; RCT for 11 and 21 due to necrosis/resorption	Extraction of 51 and 61; wire removal; FRC splinting	Repositioning of 12; FRC splinting (53–63 segment)
Follow-Up Outcome	Favorable healing; no mobility at 6 months	Stable teeth; no radiographic abnormalities at 6 months	Normal healing; stable mobility and pulpal outcomes at 6 months

Fiber-reinforced composite (FRC) splints offer controlled flexibility that supports periodontal ligament (PDL) healing while avoiding the adverse effects of rigid immobilization. The embedded glass or polyethylene fibers enhance flexural strength and distribute occlusal forces evenly, providing stability without restricting physiological mobility. Their translucency, ease of handling, and esthetics make them ideal for anterior teeth in adolescents. Flexible splints also promote functional PDL regeneration, minimizing risks of ankylosis or replacement resorption [10]. To further highlight the clinical relevance of fiber-reinforced composite splints in trauma management, their key advantages are summarized in Table 4 [7].

**Table 4:** Advantages of FRC splints This is an author-compiled summary based on Strassler and Serio [7]. FRC: fiber-reinforced composite

Advantage	Description
Better esthetics	Tooth-colored, metal-free splints provide a superior appearance, particularly important in anterior teeth of children and adolescents.
Flexibility, allowing physiological mobility	Promotes optimal periodontal ligament healing while preventing ankylosis associated with rigid splints.
Patient with metal toxicity	Suitable for patients with hypersensitivity or adverse reactions to metal alloys, as FRC splints are completely metal-free and biocompatible.
Good patient comfort and acceptance	Low-profile design improves oral hygiene accessibility, speech, and comfort.
Chairside fabrication	Easily adapted and placed in a single visit, reducing treatment time and patient burden.
Easy removal without enamel damage	Detaches cleanly from the tooth structure, minimizing iatrogenic harm during splint removal.
Less food lodgement	Smooth, contoured profile reduces plaque and food accumulation compared with wire-based splints, improving gingival health during healing

Successful outcomes depend on meticulous bonding and adaptation of the fiber, occlusal adjustment, and adherence to the IADT-recommended short splinting duration. Although pulpal necrosis may still occur, particularly in delayed presentations, regular vitality testing and radiographic monitoring enable early endodontic management to prevent sequelae. Additionally, the esthetic, low-profile design of FRC splints improves patient comfort, confidence, and oral hygiene during recovery. However, the use of FRC splints is contraindicated in situations where adequate moisture control cannot be achieved, as this compromises adhesive integrity, and in teeth with extensive crown loss that provide insufficient enamel for reliable bonding. They are also unsuitable for managing extensive alveolar fractures requiring rigid fixation rather than flexible stabilization, and in patients with poor oral hygiene due to increased plaque-retention potential of FRC surfaces. Additionally, limited crown height in mixed dentition may compromise splint stability [6].

In Case 1, the patient presented with subluxation and crown fractures that had occurred 10 days before reporting. The delay in presentation likely contributed to pulpal necrosis in tooth 11, necessitating root canal therapy, whereas tooth 21 showed external root resorption that stabilized following endodontic management. Delayed intervention has been reported to adversely affect pulpal prognosis, emphasizing the importance of early repositioning and splinting within 24 hours of trauma [3,11]. Similar findings were observed by Krastl et al. (2021), who highlighted that the degree of displacement and time elapsed before treatment are critical predictors of pulpal and periodontal outcomes [12].

In Case 2, luxation injuries involved multiple teeth with prior bridle wiring. The removal of rigid splints and replacement with FRC allowed restoration of physiologic mobility and improved oral hygiene. This outcome supports the recommendation that flexible splints should replace rigid ones to prevent ankylosis and allow PDL reorganization [6]. The patient’s follow-up revealed no residual mobility or radiographic changes, aligning with Kahler et al.'s findings that flexible splints promote functional healing and are preferable for 2-4 weeks, depending on injury severity [13].

In Case 3, repositioning of the extruded tooth and stabilization of the subluxated teeth were achieved with favourable outcomes. The decision to allow spontaneous eruption, rather than surgical repositioning, follows the principle of promoting natural eruption in immature teeth to preserve pulp vitality and minimize the risk of root resorption [14]. At the two-week follow-up, satisfactory stability was observed, consistent with IADT guidelines recommending short-term splinting for luxation injuries to facilitate biologic healing [15].

Across all cases, the use of FRC splints offered several practical advantages. They provided adequate stabilization while maintaining tooth mobility essential for PDL healing. The translucent, esthetic appearance of FRC materials improved patient acceptance, an important factor in child care. Furthermore, FRC splints allowed easy removal and minimal plaque accumulation compared to metallic alternatives. These features have been supported by Strassler and Serio, who emphasized their superior esthetics, strength, and comfort in anterior trauma management [7].

Regular follow-ups and vitality testing were crucial to detect post-traumatic complications such as pulp necrosis or external root resorption. In the present series, all splinted teeth showed favorable healing, with only one requiring endodontic intervention due to delayed treatment. This reinforces the importance of timely clinical response, gentle repositioning, and adherence to the recommended splint duration to optimize outcomes [9,13,15].

Within the limitations of this case series, all the cases showed uniform success with proper stabilization following FRC splinting. Fiber-reinforced composite splints represent a dependable, minimally invasive, and esthetically favorable option for managing subluxation in young permanent teeth in esthetic zones. However, the present case series is constrained by a short follow-up for now. The patients are still under continuous follow-up as per IADT guidelines. Future reports with larger cohorts and long-term monitoring are recommended to support these findings further.

## Conclusions

Fiber-reinforced composite splints have proven to be an effective and reliable method for managing subluxation in young permanent teeth. Their biomechanical flexibility, ease of placement, and esthetic advantage are particularly well accepted by children and adolescents. FRC enhances comfort, cooperation, and compliance, making them superior to conventional wire-based splints. This is especially valuable in pediatric dentistry, where esthetics and ease of maintenance significantly influence treatment success. In this case series, FRC splinting facilitated favorable periodontal and pulpal healing, demonstrating that short-term, flexible stabilization supports biological recovery and functional restoration. Adherence to IADT guidelines, timely intervention, and regular follow-up remain essential to achieving predictable and long-lasting clinical success.
